# Performance of two clinical fluorescence imaging systems with different targeted and non-targeted near-infrared fluorophores: a cadaveric explorative study

**DOI:** 10.3389/fvets.2023.1091842

**Published:** 2023-04-17

**Authors:** Lavinia E. Chiti, Benjamin Husi, Brian Park, Patricia Beer, Faustine D'Orchymont, Jason P. Holland, Mirja C. Nolff

**Affiliations:** ^1^Klinik für Kleintierchirurgie, Vetsuisse-Fakultät, University of Zurich, Zurich, Switzerland; ^2^Department of Chemistry, University of Zurich, Zurich, Switzerland

**Keywords:** dog, near infrared, molecular probe, surgical oncology, cat, imaging system assessment

## Abstract

**Introduction:**

Near-infrared (NIR) fluorescence-guided surgery is increasingly utilized in humans and pets. As clinical imaging systems are optimized for Indocyanine green (ICG) detection, the usage of targeted dyes necessitates the validation of these systems for each dye. We investigated the impact of skin pigmentation and tissue overlay on the sensitivity of two NIR cameras (IC-Flow^TM^, Visionsense^TM^ VS3 Iridum) for the detection of non-targeted (ICG, IRDye800) and targeted (Angiostamp^TM^, FAP-Cyan) NIR fluorophores in an *ex vivo* big animal model.

**Methods:**

We quantitatively measured the limit of detection (LOD) and signal-to-background ratio (SBR) and implemented a semi-quantitative visual score to account for subjective interpretation of images by the surgeon.

**Results:**

Visionsense^TM^ VS3 Iridum outperformed IC-Flow^TM^ in terms of LOD and SBR for the detection of all dyes except FAP-Cyan. Median SBR was negatively affected by skin pigmentation and tissue overlay with both camera systems. Level of agreement between quantitative and semi-quantitative visual score and interobserver agreement were better with Visionsense^TM^ VS3 Iridum.

**Conclusion:**

The overlay of different tissue types and skin pigmentation may negatively affect the ability of the two tested camera systems to identify nanomolar concentrations of targeted-fluorescent dyes and should be considered when planning surgical applications.

## 1. Introduction

The use of fluorescence-guided surgery (FGS) in both veterinary and human surgery has received growing interest in recent years (1–4). While traditional surgery relies on surgeons' subjective visual and tactile cues to identify target tissue intraoperatively, FGS uses a fluorescent signal to guide the surgical dissection toward the target tissue, potentially improving the sensitivity of conventional surgical techniques (1, 2, 5). Depending on the intended application, a fluorophore is administered either systemically or locally to reach the tissue of interest, and the emitted fluorescent signal is captured in real-time by dedicated camera systems. Fluorophores emitting in the near infrared (NIR) rather than in the visible spectrum are usually preferred for medical applications. The low tissue autofluorescence and reduced hemoglobin absorption in this wavelength allows for higher sensitivity and greater penetration depth (6). Indocyanine green (ICG) was the first NIR fluorophore to receive approval by the United States of America Federal Drug Administration for clinical use in human patients. To date, ICG is the most frequently utilized dye for FGS in humans and companion animals (4, 7, 8). Fluorescent lymphography with ICG has been described for sentinel lymph node biopsy in dogs with naturally occurring tumors of the bladder (9) and oral cavity (10), and has been reported in dogs and one cat with multiple mast cell tumors (11, 12). An emerging application of FGS in human medicine is the intraoperative identification of tumor margins to maximize the chances of obtaining a complete tumor excision and potentially improving the patients' outcome (13–15). Although ICG second NIR window imaging (i.e., the NIR window between 1000 and 1700 nm, in contrast to first window ranging between 700 and 900 nm) has been described for tumor visualization in surgery, the non-selective accumulation of ICG is a major limitation (1, 16). Due to this shortcoming, current approaches in NIR tumor imaging focus on the use of targeted dyes as molecular probes, that consist of fluorophores conjugated with tumor-targeted moieties. Here, the goal of improving image sensitivity by selectively accumulating the probe in tumor tissue in direct response to the expression levels of a tumor-specific biomarker (17, 18). The application of FGS and molecular probes for intraoperative identification of occult disease is still in its infancy for most tumor entities. Further investigations are encouraged due to the great potential for improvement of the current standards of care in companion animals and in humans, provided that a translational approach is adopted (1–3, 16, 19, 20). The increased usage of targeted dyes necessitates the parallel investigation of clinical imaging systems, as these are mostly optimized for ICG detection. Additionally, ICG is normally imaged at micromolar concentration, while molecular probes accumulate in the target tissue at nanomolar concentrations, raising the question on how sensitive an imaging system should be to allow for their detection.

The performance of fluorescent imaging systems for open surgery are typically tested under standard conditions and several phantom models have been developed for this purpose (21–23). Detection thresholds are objectively quantified and expressed with various parameters such as tumor-to-background ratio (TBR), signal-to-noise ratio (SNR), contrast-to-noise ratio (CNR) (24). Although this approach has the merit to produce quantitative data, it neglects the impact of the surgeon's ability to translate such data as ready-to-use information for intraoperative decision-making. Furthermore, *in vitro* models designed to mimic the optical properties of specific tissues can potentially underestimate the effect of the overlaying types of tissues, such as skin, subcutis and muscles, on the identification of the fluorescent signal. Semi-quantitative visual interpretation of images, ideally obtained under conditions closely resembling the clinical setting, are thus important to define the performances of imaging systems with different dyes and ultimately to give the end-user useful information on the reliability of camera systems for different intended clinical use.

The aim of this study was to test the diagnostic performances of two imaging systems, the IC-Flow^TM^ and Visionsense^TM^ VS3 Iridum, for the detection of various non-targeted and targeted NIR fluorophores in an *ex vivo* cadaveric model, as well as to evaluate the impact of different tissue layers and skin pigmentation on sensitivity by using both quantitative analysis and semi-quantitative visual assessment.

## 2. Methods

**Figure 1:**
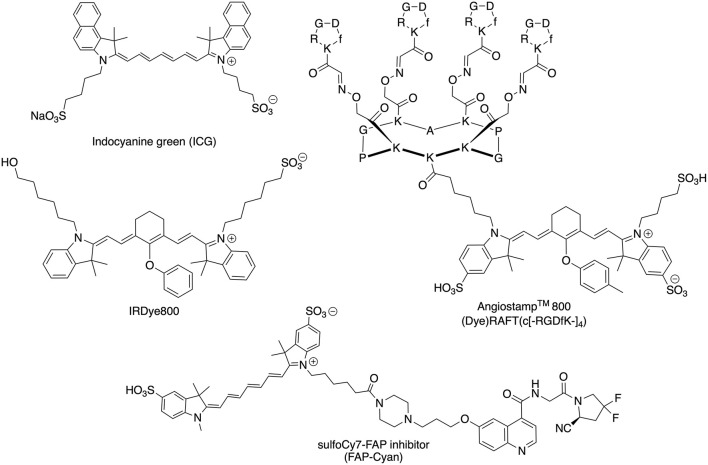
Chemical structures of the NIR dyes (ICG, IRDye600, Angiostamp^TM^, and FAP-Cyan) used in this work.

**Figure 2:**
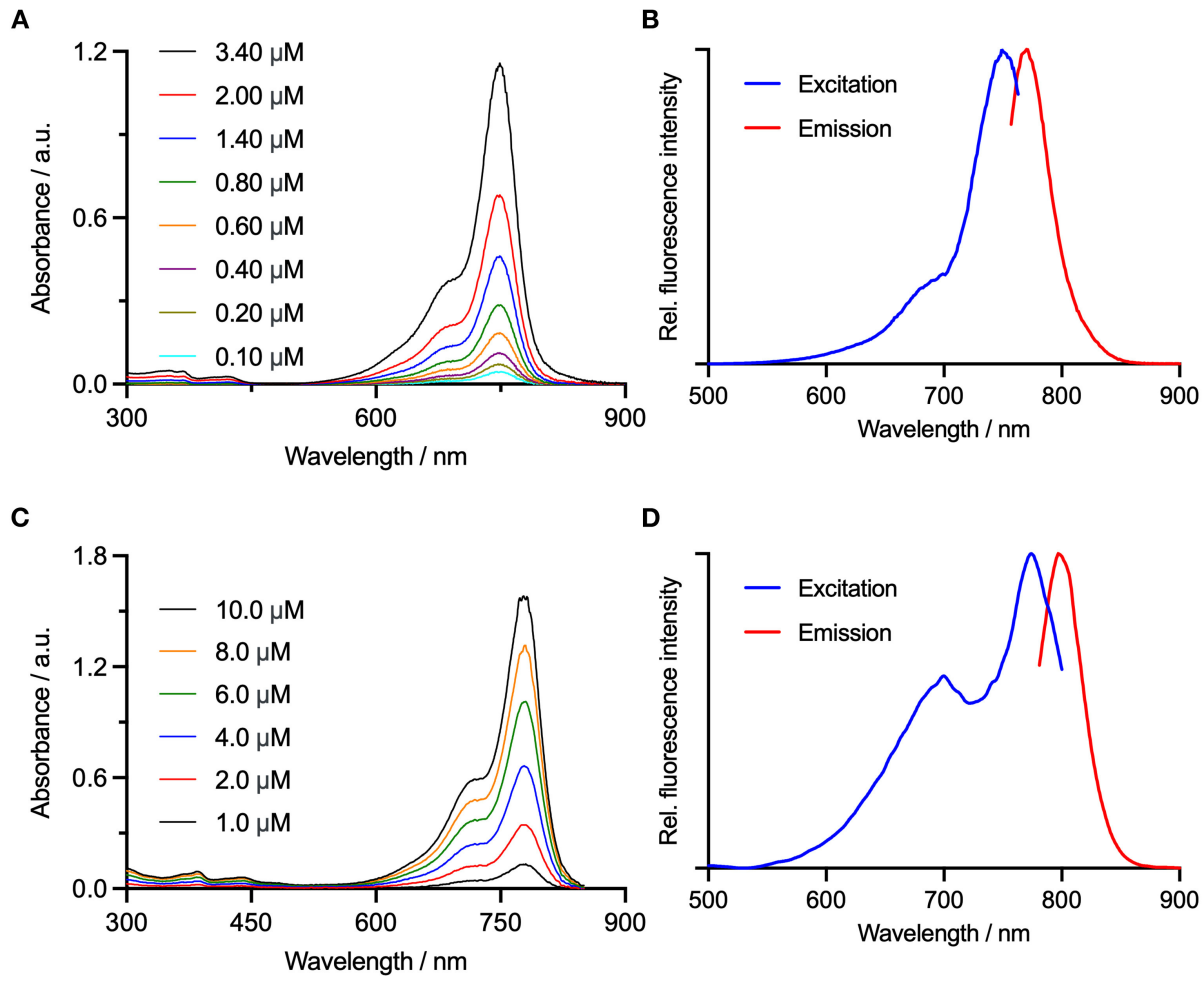
Electronic absorption spectra for **(A)** FAP-Cyan, and **(C)** Angiostamp^TM^ are shown alongside the and measured electronic excitation/fluorescence emission spectra for **(B)** FAP-Cyan, λ_ex_ = 749 nm and λ_ex_ = 781 nm, and **(D)** Angiostamp^TM^, λ_ex_ = 774 nm and λ_ex_ = 797 nm.

Two clinical NIRF cameras optimized for detection of ICG were tested: IC-Flow^TM^ (excitation peak at 740 nm, detection peak 830 nm) and Visionsense^TM^ VS3 Iridum (excitation peak 805 nm (laser), detection range 825-860 nm) (6). The cameras were tested with two non-targeted fluorophores (ICG, IRDye800), and a commercially available molecular dye binding α_v_β_3_ integrins (Angiostamp^TM^) as well as a newly developed, cyanine-7-based dye targeting Fibroblast activating protein (FAP-Cyan). Chemical structures of ICG, IRDye800, Angiostamp^TM^ and FAP-Cyan are shown in Figure 1. The measured spectra of Angiostamp^TM^ and FAP-Cyan are shown in Figure 2. Serial dilutions of ICG, IRDye800, Angiostamp^TM^ and FAP-Cyan were created with phosphate buffered saline, to obtain 6 concentrations of each dye: 10 micromolar (10 μmol/L), 1 micromolar (1 μmol/L), 0.1 micromolar (0.1 μmol/L), 10 nanomolar (10 nmol/L), 1 nanomolar (1 nmol/L) and 0.1 nanomolar (0.1 nmol/L). After preparation, the dilutions were stored in 2 mL Eppendorf tubes in dark conditions until use (for a maximum of 12 hours, to allow preparation of the cadavers).

**Figure 3:**
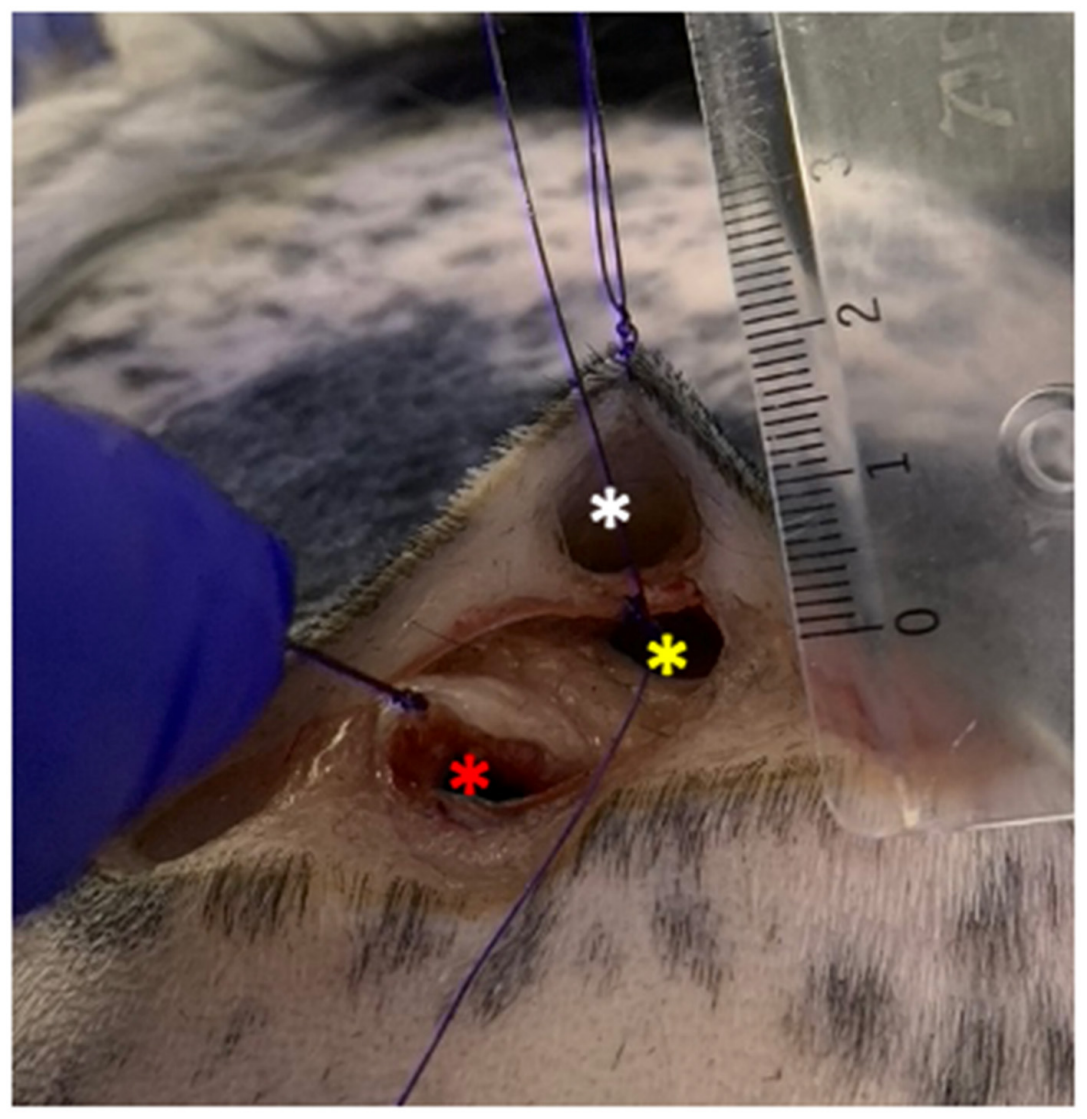
Tissue pouches on the lateral thoracic wall of the dog carcass. White asterisk: superficial pouch created right under the dermis; yellow asterisk: middle pouch created under the subcutaneous fascia; red asterisk: deep pouch created under the latissimus dorsi muscle. The pouches were all no more than 2 cm deep.

One canine and one feline cadaver were set for the experiment. The canine cadaver was a female spayed, mature, black and white-colored Cocker Spaniel and the feline cadaver was a male castrated, mature, light-colored main coon. Both animals were euthanized due to chronic non-curable and debilitating disease unrelated to the study. They were preserved in a dedicated refrigerated room at 2°C overnight and then kept at room temperature for further 12 h before the experiment. Tissue pouches were surgically created between tissue layers on the lateral thoracic wall of both carcasses: a superficial pouch directly under the skin, a middle pouch under the subcutaneous fascia, and a deep pouch under the latissimus dorsi muscle (Figure 3). The pouches were created both on an area of non-pigmented skin and on an area of pigmented skin in the dog, and on a single area of non-pigmented skin in the cat, so that when considering all the cadaver models a total of nine pouches were created.

To assess the impact of the overlapping of different tissue layers on the capabilities of the imaging systems to detect the fluorophores, the Eppendorf tubes containing decreasing dilutions of each dye were sequentially inserted one at a time in the superficial, middle, and deep pouch of each cadaver. Given that the penetration depth of NIR fluorophores ranges between 1.5 and 3 cm (20, 25) the pouches were created no more than 2 cm deep (Figure 3).

Image acquisition was conducted by a biomedical engineer employed in the field of fluorescent imaging (MK) and a postdoctoral researcher (LC) under ambient light conditions (no surgical lights, shades down, ambient room light turned on), to simulate clinical conditions of an operating theater during FGS (6). One static image was acquired for each decreasing dilution of each dye in each pouch with two imaging systems at a distance of 20 cm (ICF-Flow^TM^) or 30 cm (Visionsense VS3 Iridium^TM^) and angled perpendicular to the pouches. The Visionsense VS3 Iridium^TM^ allows for simultaneous acquisition of fluorescence and overlayed images (merge of fluorescence and white-light images), so that for each single image captured with this systems, two sets of images were produces.

**Figure 4:**
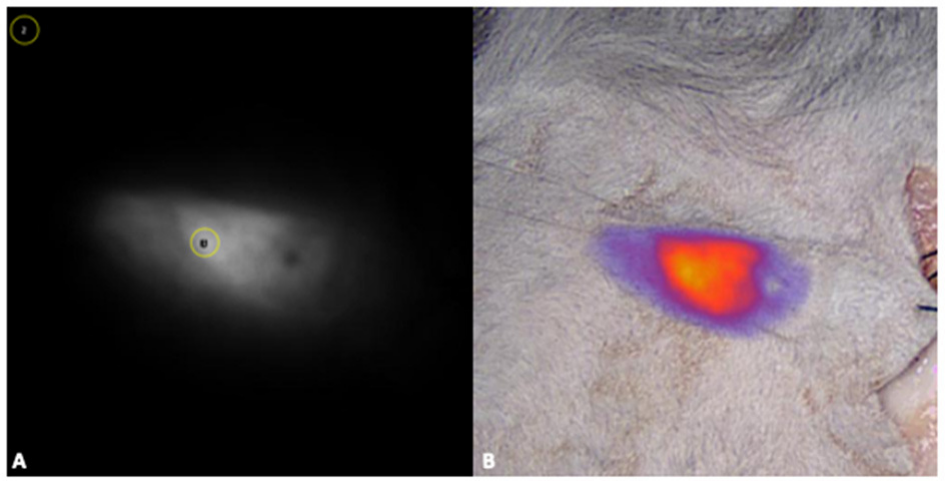
For the quantitative analysis, fluorescence images were processed with a commercially available software (ImageJ), by drawing regions-of-interest (ROI) of 30 pixels in diameter were on the most fluorescent area (1) and on a non-fluorescent area on the uppermost left corner of each image (2) **(A)**. For the semi-quantitative evaluation overlay images produced with Visionsense^TM^ V3 iridium were also used **(B)**.

The acquired images were evaluated both quantitatively and semi-quantitively. The quantitative analysis was performed on the original fluorescence images only, whereas for the semi-quantitative evaluation overlay images produced with Visionsense^TM^ V3 Iridium were also used (Figure 4). For quantitative analysis, the images were processed by a single operator (BP) using an open-source software (ImageJ). Regions-of-interest (ROI) of 30 pixels in diameter were manually drawn on the subjectively most fluorescent area and on a non-fluorescent area on the uppermost left corner of each image. The signal-to-background ratio (SBR) was then calculated by dividing the mean signal of the ROI on the fluorescent area by the mean signal of the ROI on the non-fluorescent background (26). As a measure of sensitivity, the limit of detection (LOD) of each imaging system for each fluorophore was defined as the smallest dilution at which SBR ≥ 1.5 was obtained when imaged under the superficial dermic layer (24). For each fluorophore fluorescent signal was considered non detectable with SBR < 1.5, mildly detectable with 1.5 ≤ *SBR* < 3, and clearly detectable with SBR ≥ 3 (21, 24). For semi-qualitative visual assessment, images were shown in a random order to three observers with different levels of experience: a board-certified surgeon (M.C.N.) with 3 years of experience (more than 120 procedures) with FGS, a second-year resident in small animal surgery (B.H.) with 2 years of experience assisting FGS procedures (ca. 30 procedures), and a PhD student (P.B.) with 5 years experience (ca. 60 procedures). The observers were asked to evaluate the intensity of the fluorescent signal of each image on a three-grade scale: no signal visible; signal mildly visible or very blurred; signal clearly visible. All the observers must assign one score to each image, and they were blinded to each other's score. The operator and observers are all listed as co-authors.

Descriptive statistics (median, range) was used to summarize recorded values of SBR. Kappa statistics determined the degree of agreement between the quantitative and semi-quantitative score, and the agreement between the three observers of different levels of experience. Results are reported as k weighted values and corresponding 95% confidence intervals. Interpretation of coefficients of agreement was as follows: 0.21–0.40 fair agreement, 0.41–0.60 moderate agreement, 0.61–0.8 substantial agreement, 0.81–1 perfect agreement. Level of significance was set at *p* = 0.05. Statistical analysis was performed with IBM SPSS for Macintosh version 28.0.

## 3. Results

### 3.1. Quantitative image analysis

#### 3.1.1. Limit of detection

The absolute LOD for each imaging system with each fluorophore in the *ex vivo* model is reported in Table 1. A detailed description for each fluorophore is provided below.

**Table 1:** Limit of detection (LOD) recorded for each dye, tested in each *ex-vivo* model with the two camera systems.

**Dye**	***Ex-vivo* model**	**LOD–Visionsense**	**LOD–ICFLOW**
Angiostamp	Cat	10 nmol/L	0.1 mmol/L
	Dog pigmented	0.1 mmol/L	1 mmol/L
	Dog non-pigmented	10 nmol/L	10 nmol/L
FAP-Cyan	Cat	0.1 mmol/L	10 nmol/L
	Dog pigmented	10 mmol/L	10 mmol/L
	Dog non-pigmented	10 mmol/L	1 nmol/L
ICG	Cat	0.1 nmol/L	10 nmol/L
	Dog pigmented	10 nmol/L	1 mmol/L
	Dog non-pigmented	1 nmol/L	10 nmol/L
IRDye	Cat	10n nmol/L	0.1 mmol/L
	Dog pigmented	1 mmol/L	10 mmol/L
	Dog non-pigmented	0.1 mmol/L	10 nmol/L

##### 3.1.1.1. Indocyanine green

Limit of detection of ICG imaged with Visionsense^TM^ VS3 Iridium was 0.1 nmol/L in the cat model, and at this dilution signal was mild under the skin but no longer detectable under deeper layers. In the non-pigmented dog, LOD was 1 nmol/L, and at this dilution the signal was mild under the skin and subcutis, but not detectable under the muscle. In the pigmented dog carcass LOD was 10 nmol/L, and at this dilution signal was mildly visible under the skin and no longer detectable in deeper tissue.

When IC-Flow^TM^ was tested, LOD was 10 nmol/L in the cat, with signal being mild under skin and subcutis and not visible under the muscle at this dilution. In the non-pigmented dog model LOD was also 10 nmol/L, but signal was clear under the skin and mild under deeper layers. In the pigmented dog model, LOD was 1 mmol/L and signal at this dilution was clear under the skin, mild under subcutis, and not detectable under the muscle.

##### 3.1.1.2. IRDye-800

When imaged with Visionsense^TM^ VS3 Iridium, the LOD for IRDye-800 was 10 nmol/L in the cat model, and signal at this dilution was mild under the skin and absent under deeper layers. In the non-pigmented dog model, LOD was 0.1 mmol/L with clear signal under the skin and subcutis and no signal under the muscle. In the pigmented dog, LOD was 1 mmol/L, dilution at which mild signal was visible only under the skin and subcutis and no longer detectable under the muscle.

When testing IC-Flow^TM^ with the cat model, LOD was 0.1 nmol/L and signal at this dilution was mild under all layers. In the non-pigmented dog model, LOD was 10 nmol/L with mild signal under all layers. In the pigmented dog carcass LOD was 10 mmol/L and at this dilution clear signal was visible under the skin but no signal was detected in deeper layers.

##### 3.1.1.3. Angiostamp^TM^

Limit of detection for Visionsense^TM^ VS3 Iridium in the cat model was 10 nmol/L, and at this dilution the signal was clearly visible under the skin and mild under deeper layers. In the non-pigmented dog model, LOD for this system was also 10 nmol/L, and signal was mild under all layers. In the pigmented dog model LOD for Visionsense^TM^ VS3 Iridium was 0.1 mmol/L and signal at this dilution was mild under the skin and absent in deeper layers.

With IC-Flow^TM^ a LOD of 0.1 mmol/L was recorded in the cat model, and signal was mild under the skin and subcutis and absent under the muscle at this dilution. In the non-pigmented dog LOD for this system was 10 nmol/L and signal was mild under all layers. In the pigmented dog model LOD was 1 mmol/L, and at this concentration signal was mild under the skin and no longer detectable in deeper layers.

##### 3.1.1.4. FAP-Cyan

When imaging FAP-Cyan with Visionsense^TM^ VS3 Iridium, LOD was 0.1 μmol/L, with mild signal under all layers at this dilution. Both in the pigmented and non-pigmented dog models the LOD was 10 μmol/L, and signal was clear in both models under the skin and not visible under the muscle, while under the subcutis the signal was clearly detectable in the non-pigmented model and only mild in the pigmented model. When testing IC-Flow^TM^, LOD was 10 nmol/L in the cat model and signal at this concentration was mild under the skin and no longer detectable in deeper layers. In the non-pigmented dog model, LOD was 1 nmol/L and signal was mild under all layers. In the pigmented dog model, LOD was 10 mmol/L and signal at this dilution was clear under the skin and no longer detectable in deeper layers.

### 3.2. Signal to background ratio

Measured median SBR for each camera system are reported in Table 2. Median overall SBR was lower for Visionsense^TM^ VS3 Iridium compared with IC-Flow^TM^. When considering each fluorophore separately, median SBR was higher for Visionsense^TM^ VS3 Iridium when imaging Angiostamp^TM^, ICG and IRDye 800, but was lower for FAP-Cyan. The highest mean values of SBR were obtained when ICG was imaged with Visionsense^TM^ VS3 Iridium, while the lowest median values of SBR were recorded when FAP-Cyan was imaged with Visionsense^TM^ VS3 Iridium and when Angiostamp^TM^ was imaged with IC-Flow^TM^. With respect to the *ex-vivo* models, median SBR was lower in the dog model with pigmented skin compared to the cat and dog model with non-pigmented skin, with a greater effect observed on the Visionsense^TM^ VS3 Iridium performance, although median values of SBR remained higher with this camera system compared to the other in all three models for all dyes except FAP-Cyan.

**Table 2:** Estimated median SBR (signal-to-background ratio) values for Visionsense^TM^ VS3 iridium and IC-Flow^TM^.

	**Median SBR**	**Range**
Visionsense overall	2.20	0.89–186.48
IC-Flow overall	2.68	0.64–11.57
Visionsensense (fluorophore)		
° Angiostamp ° FAP-Cyan ° ICG ° IRDye 800	3.01 1.24 7.29 2.23	0.89–163.07 0.94–186.48 0.89–155.9 0.95–148.28
IC-Flow (fluorophore)		
° Angiostamp ° FAP-Cyan ° ICG ° IRDye 800	1.96 2.66 4.35 2.33	0.78–11.08 0.88–11.57 0.77–10.52 0.64–10.79
Visionsense (*ex-vivo* model)		
° Cat ° Pigmented dog ° Non-pigmented dog	5.31 1.07 8.93	1.00–186.48 0.89–155.9 0.97–149.17
IC-Flow (*ex-vivo* model)		
° Cat ° Pigmented dog ° Non-pigmented dog	2.71 0.98 4.54	1.11–7.72 06.4–10.52 1.53–11.57
Visionsense (tissue layer)		
° Skin ° Subcutis ° Muscle	7.25 2.92 1.52	0.91–186.48 0.89–163.07 0.89–149.17
IC-Flow (tissue layer)		
° Skin ° Subcutis ° Muscle	3.55 2.42 1.95	0.78–11.08 0.64–11.57 0.65–11.49

Tissue layers also affected SBR, with progressively decreasing mean values obtained with both imaging systems for cutis, subcutis and muscle. IRDye 800 had the least decrease in median SBR between subcutis and muscle, although the highest values of mean SBR for tissue layer were always obtained with ICG.

### 3.3. Semi-quantitative visual assessment and inter-observer agreement

**Figure 5:**
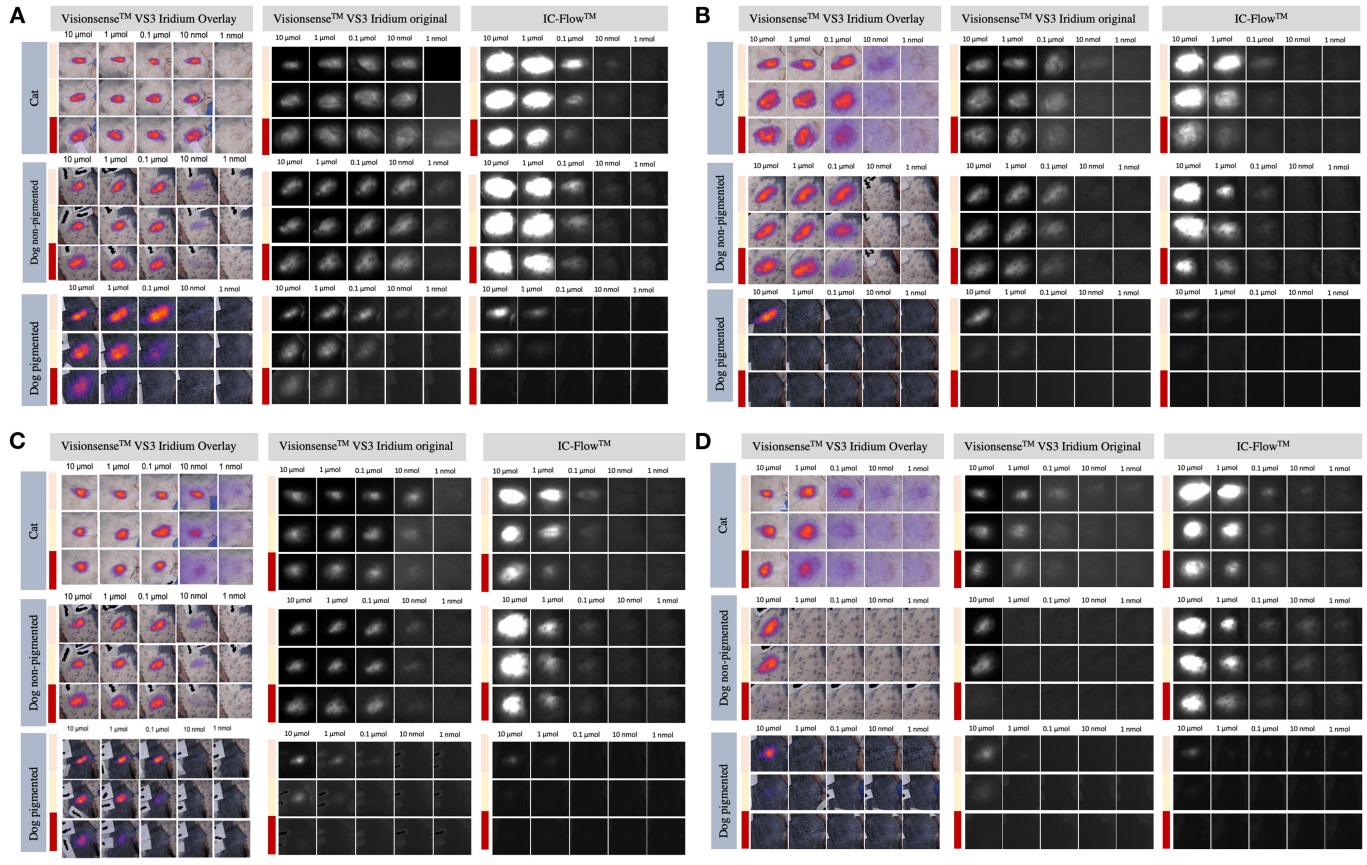
Intensity of signals from ICG **(A)**, IRDye-800 **(B)**, Angiostamp^TM^ **(C)**, and FAP-Cyan **(D)** imaged with the Visionsense^TM^ and IC-Flow systems under different tissue layers (skin pouch: beige line; subcutaneous pouch: yellow line; muscle pouch: red line).

The signals for semi-quantitative visual assessment of each concentration of each dye with each camera system are showed in Figure 5. Correspondence between semi-quantitative visual assessment of observers 1, 2 and 3 and the quantitative assessment (expressed as SBR values) are reported in Table 3. When considering the images recorded with Visionsense^TM^ VS3 Iridium, median SBR for images classified as blurred was 1.76 (range 1.35–14.24) for observer 1, 1.74 (range 1.02–3.48) for observer 2 and 1.74 (range 1.14–2.29) for observer 3. Median SBR corresponding to clear signal in the semi-quantitative scale was 49.27 (range 2.07–186.47) for observer 1, 50.7 (range 2.95–186.48) for observer 2, and 41.89 (range 1.65–186.48) for observer 3. When fluorophores were imaged with IC-Flow ^TM^, median SBR corresponding to blurred signal in the qualitative scale was 2.85 (range 0.99–6.60), 2.75 (range 0.64–9.69) and 2.29 (range 0.99–7.71) for observer 1, 2 and 3, respectively, while median SBR values for images semi-quantitatively judged as clear signal was 7.31 (range 4.35–11.57) for observer 1, 7.32 (range 4.35–11.57) for observer 2 and 6.77 (range 1.79–11.57) for observer 3.

**Table 3:** Correspondence between semi-quantitative visual score and SBR (signal-to-background ratio) measurement, for the three observers.

**Observer**	**Semi-quantitative score**	**Visionsense** ** Median SBR (min – max)**	**IC-Flow Median SBR (min – max)**
1	No signal−0	1.07 (0.89–1.44)	1.21 (0.64–7.71)
	Blurred signal−1	1.76 (1.35–14.24)	2.85 (0.99–6.60)
	Clear signal−2	49.27 (2.07–186.47)	7.31 (4.35–11.57)
2	No signal−0	1.04 (0.89–1.41)	1.19 (0.65–2.15)
	Blurred signal−1	1.74 (1.02–3.48)	2.75 (0.64–9.69)
	Clear signal−2	50.7 (2.95–186.48)	7.32 (4.35–11.57)
3	No signal−0	1.07 (0.89–1.53)	1.20 (0.64–2.26)
	Blurred signal−1	1.74 (1.14–2.29)	2.29 (0.99–7.71)
	Clear signal−2	41.89 (1.65–186.48)	6.77 (1.79–11.57)

Agreement between semi-quantitative visual assessment and quantitative analysis was perfect for Visionsense^TM^ VS3 Iridium, with k values of 0.938 (*p* < 0.001) for observer 1, 0.908 (*p* < 0.001) for observer 2 and 0.916 (*p* < 0.001) for observer 3. Agreement between the observers' evaluations and the quantitative analysis decreased to moderate with IC-Flow^TM^ for observer 1 and 2 (*k* = 0.541, *p* < 0.001) and to substantial for observer 3 (*k* = 0.663, *p* < 0.001).

When images recorded with Visionsense^TM^ VS3 Iridium were visually evaluated, interobserver agreement was perfect, with *k* = 0.887 (*p* < 0.001) between observer 1 and 2, *k* = 0.915 (*p* < 0.001) between observer 1 and 3, and *k* = 0.886 (*p* < 0.001) between observer 2 and 3. Interobserver agreement was perfect also for IC-Flow^TM^ images between observer 1 and 3 (*k* = 8.13, *p* < 0.001) while it was substantial between observer 1 and 2 (*k* = 0.806, *p* < 0.001) and between observer 2 and 3 (*k* = 0.733, *p* < 0.001).

## 4. Discussion

Fluorescence-guided surgery based on NIR fluorophores is a rapidly growing field both in human and veterinary medicine, and great interest has been raised on its potential to guide a more precise and accurate surgical dissection of neoplastic tissue than traditional “unguided” technique (3–5, 15, 18, 20, 27, 28). Indeed, implementation of NIR tracers can help the surgeon to achieve better visualization of local tumor burden, as well as to identify locoregional drainage and/or spread to the lymph nodes (1, 19, 29–31). However, several variables can affect the sensitivity of FGS, which are mainly related to the optical properties of the tissue that is imaged, the chemical characteristics of fluorophore used, the concentration of the fluorophore in the tissue of interest and the performance capabilities of the dedicated imaging systems in the selected operating conditions (6, 27, 32).

As currently most clinical NIR imaging systems are optimized for ICG, which is not used in targeted imaging approaches, it seems mandatory to test clinical imaging systems regarding their performance with different fluorophores. Moreover, the efficacy of NIR imaging is ultimately affected by the ability of the final user – the surgeon – to gather visual information for real-time clinical decision making (33). So far, very little investigation has been reported to compare the performance of different imaging systems for targeted dyes. In addition, the classic engineering which focus on camera development use different metrics to judge performance based on gold standard image analysis (6, 21, 22, 34, 35). These standardized tests are often very different from the real-world conditions in which surgeons operate. For example, computational measurements are too slow for real-time decision making and are thus not available to the surgeon during surgery. Therefore, it is essential to evaluate if subjective visual judgement of the surgeon corresponds to standard measurements. In the study presented here, we created an *ex vivo* model to resemble a typical clinical setting where FGS could be employed, and we evaluated how the above-mentioned variables affect the performances of two fluorescent imaging systems designed for open imaging–the VisionSense^TM^ VS3 Iridium and IC-Flow^TM^.

For both camera systems, the best performance in terms of SBR and the LOD were achieved when imaging ICG. This result is not surprising, given that both imaging systems have been optimized and approved for clinical use with this fluorophore. However, ICG is not the most favored fluorophore for targeted imaging due to several limitations (1, 4, 16). The emission and excitation spectra of targeted dyes differ from ICG due to addition of ligands and/or the usage of a different fluorophore backbone. At the same time, they are imaged at nanomolar concentrations (3, 14, 33). This requires evaluation of the imaging systems with respect to individual dyes at different concentrations.

In the present study, Visionsense^TM^ VS3 Iridium was able to detect nanomolar concentrations of both Angiostamp^TM^ and IRDye 800 in the cadaveric cat model and of Angiostamp^TM^ in the non-pigmented dog carcass, whereas with IC-Flow^TM^ nanomolar concentrations of these fluorophores were only visible in the non-pigmented dog carcass. The fact that the imaging systems were able to detect these fluorophores at such concentrations in the tested cadaveric model suggests that, despite the original approval being issued for ICG only, they could potentially be successfully used for target fluorescent imaging with the tested dyes. However, this consideration needs further confirmation in future studies on live animals undergoing proper intravenous administration of the fluorophores.

The impossibility to detect signal at nanomolar dilutions in the pigmented dog carcass and the decreased values of SBR recorded in the latter *ex vivo* model raise questions on the reliability of transdermal NIR imaging in the nanomolar range in patients with pigmented skin. It is well known that the optical properties of tissues can affect the performances of FGS, mainly due to absorption and scattering of photons from tissue components such as hemoglobin, water, and lipids (27, 32). Melanin is as a minor absorber and its presence in living tissues at different concentrations can act as a superimposed filter thus altering the absorption spectrum (36, 37). Since, melanin mainly absorbs light in the visible spectrum, signal attenuation when using NIR imaging should theoretically be minimal. However, in a study on a murine model NIR fluorescent signal was significantly impacted by depilation-induced skin pigmentation, with NIR fluorescent signal amplitude being reduced up to 39% in heavily pigmented compared to non-pigmented mice (38). Likewise, in the present study we demonstrated that natural dark skin pigmentation in a large animal model negatively affects the signal from different NIR fluorophores (32). Pigmentation of the skin should thus be considered when planning to implement FGS, especially if very low thresholds of fluorescence are to be expected. Further investigations on the absorption properties of increasing concentrations of melanin and its interaction with other skin pigments in different species are warranted to better understand to which extent skin pigmentation can affect the reliability of FGS in applications where transdermal imaging is relevant.

The Visionsense^TM^ VS3 Iridium system outperformed the IC-Flow^TM^ for three of the four tested fluorophores in this experiment, both in terms of sensitivity to lower concentrations of fluorophores and SBR (ICG, IRDye-800 and Angiostamp). In accordance with our results, in a recent review investigating the performance capabilities of six different fluorescent imaging systems, the highest values of sensitivity to low concentrations of IRDye 800CW and IRDye 680RD were also recorded for the Visionsense^TM^ VS3 Iridum, due to favorable technical characteristics such as camera bit depth and gain adjustment (6). IC-Flow^TM^ outperformed the Visionsense^TM^ VS3 Iridum when FAP-Cyan was imaged. This observation can be explained due to better overlapping between the excitation spectrum of FAP-Cyan and emission spectrum of the laser of IC-Flow^TM^ compared to Visionsense^TM^ VS3 Iridium. It also underlines the above statement, that imaging systems' performances are closely related to the dye in use.

Finally, the superimposing tissue must be considered, especially in FGS tumor imaging. Although the NIR window allows for deeper penetration into tissues compared to other wavelengths, the maximum depth for signal detection ranges among 1.5 and 3 cm (4, 20). Depending on the anatomical region, different tissue layers and tissue types, with variable optical properties, can be encountered within 3 cm from the body surface. It is thus reasonable to assume that the intensity of fluorescent signal would be affected not only by depth, but also by the composition of the different tissue layers that overlay the target tissue. In the study presented here, the impact on fluorescent signal of three different overlying tissue types, namely the dermis, dermis plus subcutis and dermis plus subcutis plus muscle, was evaluated. As expected, detection sensitivity decreased with increasing depth for all dyes and both imaging systems. When placed under the muscular layer, SBR decreased and fluorescent signal was not visible anymore at LOD dilution in most cases. Given that the muscle is rich in hemoglobin, which is well-known to cause absorption in the NIR wavelength, it seems reasonable to hypothesize that if the target tissue is located under the muscular fascia, the resulting fluorescent signal could be impaired by the composition of this tissue layer in addition to depth (27, 32).

The impact of a surgeon's experience with FGS on the sensitivity of the procedure was assessed by asking to three observers with different levels of expertise to evaluate the images produced with Visionsense^TM^ VS3 iridium and IC-Flow^TM^. Agreement between the observers was perfect to substantial with both camera system. The low interobserver variance that we report suggest a high reliability of the technique, also for operators at the beginning of the learning curve. Interestingly, when the scores of the observers were compared to the gold standard, it resulted in perfect agreement for the Visionsense^TM^ VS3 Iridium, while agreement was only moderate to substantial for IC-Flow^TM^. This observation roots in a higher variance between semi-quantitative visual assessment and quantitative score for images taken with the latter system. It could be hypothesized that images produced with Visionsense^TM^ VS3 Iridium, which have higher resolution compared to IC-Flow^TM^, are easier to be interpreted by the operator, thus leading to less variance between the objective quantitative score and the subjective visual assessment (6). This observation is also supported by the higher median values of SBR recorded for Visionsense^TM^ VS3 Iridium and underlines the reliability of this imaging systems for open imaging.

A strength of this study is that variables that may affect sensibility of fluorescent imaging systems in a clinical setting, such as pigmentation of the skin and type of layers covering the tissue of interest have been included. To the best of our knowledge corresponding studies have not been performed before. Furthermore, the use of both a quantitative image analysis system and a semi-quantitative visual score allowed to evaluate the impact of surgeon's interpretation of the images on the overall sensibility of the procedure.

The main limitation is that images were acquired only on two cadavers, thus potentially neglecting the variability between individuals. The decision to include only two cadavers was mainly related to the explorative nature of the study. In future studies, cadavers of different dogs and cats breeds, of individual with different nutrition status and of varying ages should be included to evaluate the impact of intraindividual variability on the tested variables. Another limitation is that the present study was conducted *ex-vivo*, thus not allowing for the evaluation of the interactions between the fluorophores and the tissue components that normally occur in the living animal. The results of this study should encourage further investigation of the performances of different camera systems with targeted and non-targeted fluorophores on live animals after local or intravenous administration of the fluorophore of interest. Lastly, the study design did not allow to perform a multivariate statistical model to evaluate the conjunct impact of variables on the sensitivity of the two imaging systems.

## 5. Conclusion

In conclusion, based on the result of this explorative study, Visionsense^TM^ VS3 Iridium seems to performed better than IC-Flow^TM^ for the detection of small dilutions of the tested non-targeted and targeted fluorophores, with the exception of FAP-Cyan. When the semi-quantitative visual score was compared with the quantitative image analysis, variance was lowest for Visionsense^TM^ VS3 Iridium, suggesting that subjective interpretation of images produced with this system could be more reliable than interpretation of IC-Flow^TM^ images.

## Data availability statement

The raw data supporting the conclusions of this article will be made available by the authors, without undue reservation.

## Ethics statement

Ethical review and approval was not required for the animal study because the experiment was non conducted on live animals, but on cadavers of animals that had been euthanised due to medical reasons unrelated to the study. Written informed consent for participation was not obtained from the owners for the participation of their animals in this study was not required in accordance with the national legislation and the institutional requirements, as no live animals were used. Owners, however, signed a written consent for use of their animals' cadavers for scientific purposes after euthanasia performed for medical reasons unrelated to the study.

## Author contributions

Conception and design of work: MN and LC. Acquisition and analysis of data: LC, MN, BP, BH, PB, FD'O, and JH. Interpretation of data: LC and MN. Drafting of the manuscript: LC. Substantial revision of the manuscript: MN and JH. All authors have approved the submitted version and have agreed to be accounted for contribution.
